# Enhancing Aotearoa, New Zealand's Free Healthline Service through Image Upload Technology

**DOI:** 10.1155/2024/6644580

**Published:** 2024-02-02

**Authors:** Miriama K. Wilson, Fiona Pienaar, Ruth Large, Matt Wright, Verity F. Todd

**Affiliations:** ^1^Paramedicine Research Unit, Department of Paramedicine, Faculty of Health and Environmental Sciences, Auckland University of Technology, 649 Great South Road, Manukau, Auckland 2104, New Zealand; ^2^Whakarongorau Aotearoa/New Zealand Telehealth Services, Auckland, New Zealand

## Abstract

**Background:**

Healthline is one of the 39 free telehealth services that Whakarongorau Aotearoa/New Zealand Telehealth Services provides to New Zealanders. In early 2021, an image upload system for viewing service user-uploaded images was implemented into the Healthline service.

**Aims:**

The aim of this research was to understand the utilisation of Healthline's image upload system by clinicians and service users in New Zealand.

**Methods:**

This is a retrospective observational study analysing Healthline image upload data over a two-year period: March 2021 through to December 2022. A total of 40,045 images were analysed, including demographics of the service users who uploaded an image: ethnicity, age group, and area of residence. The outcome or recommendation of the Healthline call was also assessed based on whether an image was included.

**Results:**

Images uploaded accounted for 6.0% of total Healthline calls (*n* = 671,564). This research found that more service users were advised to go to an Emergency Department if they did not upload an image compared to service users who used the tool (13.5% vs. 7.7%), whereas a higher proportion of service users were given a lower acuity outcome if they included an image, including visiting an Urgent Care (24.0% vs. 16.9%) and GP (36.7% vs. 24.3%).

**Conclusion:**

Service users who did not upload an image had a higher proportion of Emergency Department outcomes than service users who did use the tool. This image upload tool has shown the potential to decrease stress on Emergency Departments around Aotearoa, New Zealand, through increased lower acuity outcomes.

## 1. Introduction

Healthline provides a health triage service to people living in Aotearoa, New Zealand, enabling access to health advice 24 hours a day, seven days a week, using a free-to-call phone number [1]. Healthline is funded by the New Zealand Government with the purpose of giving trained health advice to all New Zealanders for free regarding where they should go to receive the appropriate treatment based on their health symptoms. This service hopes to reduce the number of patients presenting at Emergency Departments around Aotearoa with nonserious health conditions and therefore reduce hospital overcrowding. Healthline is the only free telehealth service available in New Zealand, available any time of the day or night, to anyone. This line is staffed by telehealth-trained clinicians (paramedics and nurses) who utilise a clinical decision support tool to assist in advising patients with either self-care advice or when and where to seek further medical attention [1]. The ability to upload images by service users during the phone call was introduced to Healthline in 2021. This was undertaken to augment the clinician's ability to give timely advice, particularly concerning wound care and skin conditions.

Additional visual information for skin conditions via the adoption of advanced digital and mobile phone technology has resulted in a growth in the use of high-quality smartphone images [2–4]. However, relying on service users to upload images has revealed some challenges, including compromised patient care due to a lack of image quality to allow for accurate interpretation. Additionally, poor digital literacy and disinterest in using technology create barriers to user uptake. There are also indications that the increased engagement from involving service users in the transfer of images leads to more purposeful discussion and better management of their health, therefore improving health outcomes from their increased knowledge and awareness [3].

The introduction of an image upload system to telehealth was seen internationally in response to the COVID-19 pandemic to reduce the number of patients presenting at Emergency Departments [5]. The benefits of an image upload are the ability to revisit an image from a patient rather than a one-off glimpse by a specialist and shorter consultation times [5]. Telehealth services have the potential to decrease health inequity, especially for Māori and rural communities in New Zealand [6]. Through this research, we hope to show the benefit of image upload technology through a free telehealth service that caters to all health symptoms. This in turn will provide beneficial evidence to allow for the adaptation of an image upload system to other heavily image-dependent specialties including ophthalmology in Aotearoa, New Zealand. Literature has shown internationally that an image upload system is accurate and beneficial for monitoring skin conditions and injuries [7–10].

The aim of this research was to understand the demographics of service users using Healthline's image upload tool, including ethnicity, age group, and urban-rural profile, as well as the uptake of the tool by Healthline clinicians. This study analysed the call outcomes (recommendations) made through Healthline to determine the impact on caller outcomes when an image was uploaded.

This research is relevant and novel as this is the first time an image upload system has been introduced to a free telehealth service in New Zealand. This service was introduced to increase health outcomes for New Zealanders at no cost to patients, especially in demographics that are disproportionately affected by severe skin conditions, including Māori and Pasifika ethnic groups and rural communities [11].

## 2. Methods

### 2.1. Study Design

This is a retrospective observational cohort study using routinely collected telehealth data to assess the outcomes in service users utilising image upload. Service users are defined as any person who calls any of the free 39 telehealth services provided by Whakarongorau Aotearoa. For this study, service users refer to the participants who called the free Healthline service (between 1 March 2021 and 31 December 2022) and uploaded an image on request of the call handler.

### 2.2. Setting

Whakarongorau Aotearoa/New Zealand Telehealth Services (Whakarongorau) is funded by the New Zealand Government, making all their services free and available 24/7 via phone, text, and web chat [12]. In March 2021, Whakarongorau implemented an image upload system to their free 24/7 Healthline service. An image is requested by a clinician when they would like more information on any superficial condition (e.g., rash, wound, or swelling) in order to give a better-informed health recommendation. It is entirely up to the call handler/clinician whether they choose to use the image upload service; however, it is encouraged by the Healthline clinicians. The clinician/call handler will ask the service user to take and send a photo using a link sent through by text message. This image is uploaded directly to their clinical note system; the image will then be stored in the service users' confidential clinical records. There is no cost to the service user when uploading an image. These records can be connected to a service user's NHI (National Health Index) number. This allows Healthline clinicians to access a service user's previous health records to provide the best-informed advice. The image uploaded must only be of the relevant body part and should not allow for physical identification of the service user. This is to protect the privacy of the service user in the case of the photo being shared between clinicians. The clinician is required to ask for consent, when possible, for this image to be included in the health assessment. The clinician can then assess the image and give the appropriate advice with the help of the Odyssey (Advanced Computer Software Group Ltd., United Kingdom) question tool [13], an internationally accredited decision support tool. The clinician's role is not to diagnose but to give appropriate, timely advice to a service user. These images are not shared elsewhere, meaning they are not included in consult notes sent to the service user's general practitioner (GP). However, the GP can read that an image upload was used in the health assessment and follow up with the patient to see the image on their phone, if needed.

Service user data was collected and analysed as previously described [11], with data provided in a deidentifiable form. Image upload data from service users was collected between 1 March 2021 and 31 December 2022 (22 months). STROBE cohort reporting guidelines were followed [14].

This research project was approved by the Auckland University of Technology Ethics Committee (AUTEC) (23/28).

### 2.3. Participants

The data included in this research project involved service users who called the free Healthline service on 0800 611 116 and uploaded an image to the Healthline system based on the advice of the Healthline call handler. For this research, image upload contacts refer to the number of images uploaded, where multiple images could be uploaded by the same service user (on one or numerous occasions). Individual contacts or images refer to the number of individual (or unique) service users using the service.

### 2.4. Variables

Deidentified data were provided for age group, ethnicity group (prioritised) [15], and rural-urban profile (using GCH data) [16], as Wilson et al. described [11]. Prioritised ethnicity is used in accordance with data collection in the New Zealand Health and Disability sector where only one ethnic group was recorded. This avoids issues including multiple data points for one service user. When service users call Healthline, they may be asked to upload an image. After uploading this image, the clinician advises the service user on what they should do based on their symptoms. This is known as the call outcome; call outcomes range from the most acute outcome, “111 Emergency,” where an ambulance is called for the user, to the least acute outcome, “self-care,” and to call back if they have any further concerns [11]. Other outcomes included in this research include advising a service user to go to an Urgent Care clinic, stay on call to speak with a Whakarongorau doctor/general practitioner (GP), book an appointment with their GP, and go to a pharmacy to speak with a pharmacist. The “other” outcome is used when a service user calls in asking for general information rather than health advice, or they have hung up/got the wrong number [11]. The “on-call doctor” outcome is often used when a service user is unable to book an appointment with their GP in the near future, usually due to their GP being booked out for weeks or not being enrolled with a GP practice. This research does not show whether a service user follows the advice and attends a healthcare service such as an Emergency Department.

Whakarongorau Aotearoa's Healthline service is not responsible for diagnosing but for acting as a triage service to recommend a service user based on their symptoms and where to go to receive the appropriate treatment. As this service is not diagnostic, the recommendation or call outcome depends on several factors including symptoms, location, time of day, and the location of the nearest healthcare provider. If a service user states they cannot afford a service such as Urgent Care, this is factored into their call outcome. The only options for care are an Urgent Care clinic and Emergency Department if a service user calls after 6 p.m. on any given day [11]. In certain rural areas, there may be on-call general practitioners available 24/7.

Clinician uptake is described in this research and refers to the number of clinicians or call handlers (used interchangeably) who used the image upload service (in a given month). This means they requested a service user to upload an image using a link sent by the clinician, allowing the clinician to see the image and store it in the service user's health records (if appropriate).

### 2.5. Statistical Methods

R and RStudio were used for statistical analysis (including chi-square, ANOVA, and linear regression) [17].

A one-way ANOVA test was conducted to determine statistical significance in the total number of image contacts per day per month. A chi-square proportional test was used to compare proportions for gender, age, ethnicity, rurality, and outcome data, compared to the total Healthline contacts. A simple linear regression model was used to determine a linear relationship with clinician uptake over time. Differences with value (*p*) < 0.05 (^∗^) were deemed statistically significant [17]. Levels of significance are represented by an asterisk (^∗^), with ^∗∗∗^ being the highest level of significance (*p* < 0.001(^∗∗∗^)) and then *p* < 0.01(^∗∗^) and *p* < 0.05(^∗^) being the lowest level but still significant [17].

## 3. Results

Between 1 March 2021 and 31st of December 2022, a total of 40,045 images were uploaded to Healthline by service users, with an average of 59.9 uploads per day each month, of which individual service users uploaded 39,256 images (98%). This means that 789 images were duplicates or multiple images uploaded by the same service user; this, however, only accounts for 2.0% of images uploaded during this time. In 2021, 16,419 (16,097 individual) images were uploaded; in 2022, there were 23,626 (23,159 individual) image uploads. The highest month for image upload contacts was December 2022, with 2,720 uploads, averaging 87.7 daily uploads. Image upload contacts have increased with time, peaking on Sundays and between six and seven p.m. daily. The service user symptoms that resulted in the highest number of image uploads included rashes (10,169, 27%), wounds (1,946, 5%), bites/stings (1,755, 5%), immunisations (902, 2%), and itch (811, 2%). Image upload data was compared to total Healthline calls during this period (671,564 calls); images accounted for 6.0% of total Healthline calls. Mid Central District had the highest number of image uploads per 10,000 people, with 100.9 image uploads per 10,000 people (2021-2022).

### 3.1. Image Upload Contacts by Month

Healthline image upload contacts were investigated per day (by month) in 2021 and 2022. Mean value is 59.9, median value is 59.0, and IQR is 28.0. See supplementary material (Table T1) for more information.

**Figure 1:**
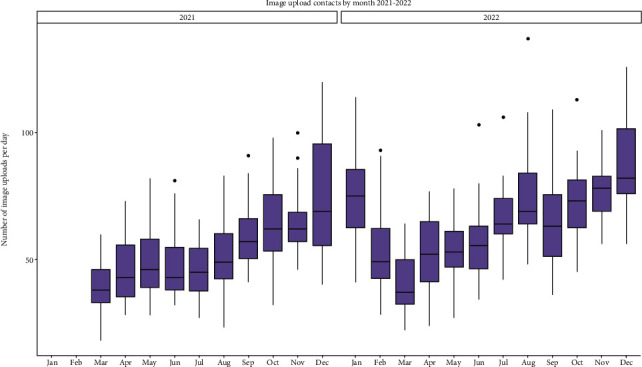
Side-by-side box and whisker graphs comparing the total number of image upload contacts per day, by month, in 2021 and 2022.

**Figure 2:**
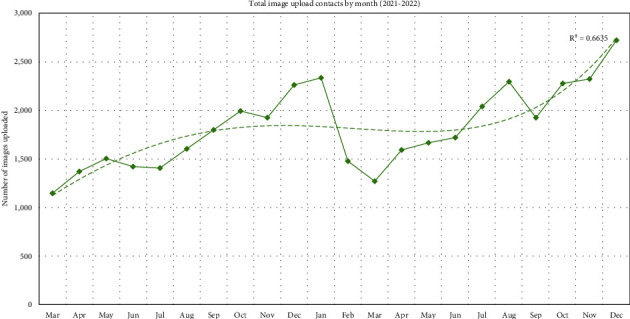
Total image upload contacts for each month 2021-2022, with a trendline.

As shown in Figure 1, there is an apparent increase in images uploaded as the year goes on, with December having the highest image contacts in 2021 and 2022. March had the lowest number of images uploaded in 2021 and 2022; however, the image upload service was only implemented in March 2021. There was a significant difference between images uploaded by month of the year (*p* < 0.001(^∗∗∗^)). The average image upload contact per day was 59.9, with a median of 59.0 and an interquartile range (IQR) of 28.0.  Figure 2 represents the total images uploaded each month and the increasing trend in image uploads with time even with the dip in uploads in March 2022. Image uploads increased over time from 1,146 in March 2021 to 2,720 image uploads: a 2.4-fold increase in 22 months.

### 3.2. Image Upload Contacts by Ethnicity

The number of Whakarongorau image upload contacts was investigated by service users' demographics, including gender, age group, prioritised ethnicity, and rurality in 2021 and 2022, as shown in Table 1.

Of the 40,045 images uploaded, 21% were uploaded by Māori (8,319), and 6% were by Pasifika (2,284) (Table T2, supplementary material). Māori are overrepresented in the image upload data compared to the total Healthline contact proportion (17.7%) (*p* < 0.001(^∗∗∗^)); this was also observed in NZ European and Asian ethnic groups. As shown in Table 1, there are multiple statistically significant differences in proportions for the demographic data for total Healthline contacts and image upload contacts. The unknown or anonymous data should be acknowledged as a limitation, meaning the results should be interpreted with caution.

### 3.3. Image Upload by Age

**Figure 3:**
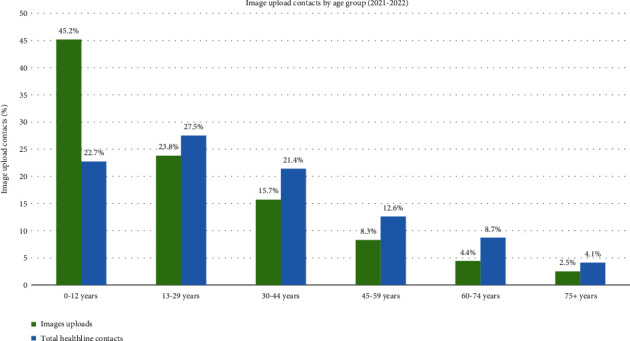
The proportion of image uploads and total Healthline contacts by age group from 2021 and 2022. See supplementary material, Table T3.

Image upload contacts were investigated by age group in 2021 and 2022. The highest number of images uploaded was regarding service users aged under 13 years, with 18,103 or 45.2% of the total image upload contacts, as shown in Figure 3. The age group with the lowest image upload contacts was service users over 75 years, with 1,001 or 2.5% of the total image upload contacts (seen in Figure 3). In comparison, the highest Healthline calls are regarding 13–29-year-olds with 27.5% of calls and the lowest from over 75 years with 4.1% of calls (see supplementary material, Table T3). In total, 52.7% of the image upload contacts were for service users under the age of twenty, whereas 30.3% of Healthline contacts were regarding under 20-year-olds. A chi-square proportional test proved significant differences in proportions for each age group when compared image upload to total Healthline contacts.

### 3.4. Image Upload Contacts by Rurality

Healthline image upload contacts were investigated by their New Zealand urban-rural profile in 2022 (see supplementary material: Figure S1, Table T4, and T5). In total, 78.7% of image upload contacts come from service users located in urban areas, and 14.8% are from rural areas (with 6.5% unknown). This is comparable to New Zealand's urban and rural statistics, with 16.3% of New Zealanders living in rural areas and 83.7% in urban areas [18]. Statistical analysis proved no significant difference in the proportion of images uploaded by rural contacts compared to the NZ rural population (excluding the unknown group).

### 3.5. Image Upload Contacts by Outcome

**Figure 4:**
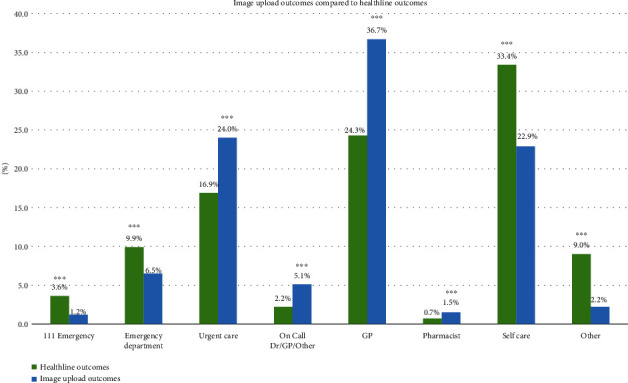
Side-by-side bar charts comparing the service user outcomes for total Healthline calls and Healthline calls that included an image upload (2021-2022). Levels of significance are represented by an asterisk (^∗^), with ^∗∗∗^ being the highest level of significance (*p* < 0.001(^∗∗∗^)) and then *p* < 0.01(^∗∗^) and *p* < 0.05(^∗^) being the lowest level but still significant [17]. Significantly higher proportions are represented with an asterisk (^∗^). See supplementary material, Table T6.

Healthline image upload contacts were investigated by call outcome (2021-2022), compared to all Healthline contact outcomes for the same period, as shown in Figure 4.

Figure 4 shows an apparent difference in call outcomes for service users who upload an image compared to no image uploaded. Healthline call outcomes with images uploaded only make up 4.9% (*N* = 671,564) of the total Healthline call outcomes (due to unknown call outcomes for 7,145 images uploaded). This data shows “111 Emergency” and ED; the highest acuity outcomes have significantly lower proportions than the total Healthline call outcomes (*p* < 0.001(^∗∗∗^)). Urgent Care and GP (proportion) outcomes are significantly higher for the image upload outcomes (*p* < 0.001(^∗∗∗^)). It is inferred that the significantly lower proportion (*p* < 0.001(^∗∗∗^)) for the “self-care” and “other” outcomes for images uploaded is due to an image upload being unlikely to be necessary. The “other” outcome is given for calls regarding general information or a wrong number/hung-up call, meaning an image is not necessary. The self-care outcome is given for general health information inquiries as well as symptoms that can be managed at home, such as with paracetamol or a plaster/bandage.

### 3.6. Clinician Uptake of Image Upload Service

The uptake of the image upload service by Healthline clinicians was investigated by month (2021-2022).

**Figure 5:**
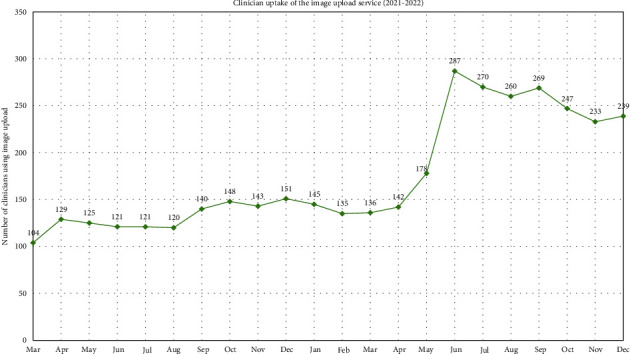
The number of clinicians utilising the image upload service by month (Mar 2021-Dec 2022). See Table T7 in supplementary material.

Figure 5 shows that clinician uptake of the image upload service has changed with time, with the highest number of clinicians using the service in June 2022 and the lowest in the first month of the service (Mar 2021). In a two-month period (Apr-June 2022), clinician uptake more than doubled from 142 to 287 clinicians using the service. A simple linear regression model was fit to data comparing clinician uptake (per month) with image uploads (per month); the trendline produced had a significant *p* value of 0.0023 (^∗∗^) (see supplementary material, Figure S2). This represents strong evidence of an increasing trend in image uploads by service users when there is higher image upload uptake by clinicians.

## 4. Discussion

Over this period, 6.0% of service users who called Healthline used the image upload tool. These service users had a higher proportion of lower acuity outcomes including “Urgent Care,” “on-call Dr,” and “GP,” than service users who did not upload an image. A possible explanation for the “GP” outcome being the most common outcome (36.7%) for image upload data is due to the need for a common GP prescription of an antimicrobial ointment or pill for a service user's visible symptoms. Furthermore, service users who did upload an image had a lower proportion of Emergency Department outcomes compared to service users who did not use the tool. However, from analysing clinician uptake data of the image upload tool, it is apparent that not all clinicians choose to use the tool, although the use has increased with time. The large increase in clinician uptake (and peak) corresponds with a Healthline campaign to increase the number of clinicians using the image upload tool. This is an interesting finding, as the image upload tool potentially provides a clinician with more information on health symptoms and may provide more visual information than if an image was not included. This tool has the potential to result in fewer ED outcomes for service users, which could alleviate stress on already overcrowded Emergency Departments around Aotearoa.

Although image upload outcomes resulted in fewer ED recommendations, not all service user's symptoms are superficial, with symptoms such as chest pain needing immediate medical attention and likely one of the reasons total Healthline call outcomes have a much higher proportion of ED outcomes. In addition, suspicion of a high-acuity concern may prompt a clinician to bypass uploading an image for a symptom that clearly needs immediate attention. An example of this is when the simple “glass test” is performed by a Healthline caller, which does not necessarily require an image upload. This test indicates whether a service user has a purpuric or petechial rash, which requires further investigation as it may warrant sepsis and disseminated intravascular coagulopathy complications; an example of this is meningococcaemia, a disease of concern in New Zealand [19]. This quick test results in an immediate ED outcome, meaning an image upload is unnecessary.

From analysing the image upload data for Healthline calls, it appears that Māori are overrepresented in this data (21%) when compared to their NZ demographic (17%) [20]; this is consistent with our previous findings [11]. This is a positive finding that Māori are using the image upload tool to allow for better-informed decisions on their health. However, Pasifika are underrepresented; this is unexpected but also consistent with our previous findings [11], as Pasifika, similar to Māori, have high numbers of severe skin conditions presenting at ED around Aotearoa. Image uploads peak in December and trough in March for Healthline, which coincides with the New Zealand summer holidays in December (ending in February), resulting in more injuries and New Zealanders having more time to prioritise their health [21]. Certain skin conditions are also more likely during the summer period due to hot weather, causing sweating and an increased likelihood of insect bites or stings [22]. This also coincides with the closure of medical clinics during the shared Christmas and New Year's holidays, meaning regular healthcare practices are unavailable. The highest image uploads were for service users aged between two and five years, with over 65-year-olds only contributing to 5% of the image uploads. This is not unexpected, as older generations have shown to be less comfortable with smartphone use and thus an attached camera [7]. This also could be due to these populations being less likely to present with health conditions that require photos. There was no apparent relationship with rurality, which is unexpected as rural patients have more barriers to accessing primary care in New Zealand [11].

International literature has found positive results with the use of an image upload telehealth system for the treatment and monitoring of skin conditions [7–10]. The low uptake of image upload in patients over 65 is consistent with previous research; for example, in discharged cellulitis patients with telehealth follow-up, 81% of patients under 65 uploaded an image, compared to only 31% of patients over 65 [7]. Image screening has been effectively demonstrated in dermatological studies, with 70-95% agreement between the image screen and in-person consults [8–10]. These findings suggest that image upload is a good alternative for in-person clinician consultations.

The limitations of a telehealth image upload system include image quality, lighting (especially at night), positioning, and Internet access, some of which are ultimately determined by the device used by a service user and the accessibility of a device. Healthline clinicians are, however, taught how to improve an image within the constraints of the device being used. Examples of this include optimisation of lighting such as using flash on a device, centring the image, and encouraging the service user to take more than one image and sending in the image with the best quality. The dependency of image upload quality on device availability and connectivity disadvantages lower-income earners and rural patients [23]. Other issues include language barriers, technical difficulties, the depersonalisation of medical care, patient consent, and the privacy and security of images uploaded (including their use in a court of law) [23]. There is also the issue of a service user calling in from a landline phone or phones without cameras, making image upload impractical.

The obvious advantage of a telehealth image upload system for New Zealanders is that they can access free 24/7 healthcare advice from well-informed, trained health professionals compared to international countries where image upload health advice comes at a cost to the service user [23]. Telehealth was an essential service during the COVID-19 pandemic, with limited availability of GP appointments and Emergency Departments overrun with patients infected with COVID-19 [24]. Image upload technology is able to better support Healthline and has been shown in this research to reduce the number of service users being advised to go to ED. This is a significant finding, as EDs across Aotearoa are already stressed and overcrowded. Through image upload, service users are better informed on what to do to receive the appropriate treatment, resulting in better health outcomes.

This research has shown the benefit of an image upload system to a telehealth service through increased lower acuity call outcomes, potentially reducing the number of patients sent to Emergency Departments around New Zealand. An image upload service allows for potentially better-informed health advice by Healthline clinicians, where the future adoption of a similar system to other health services could prove beneficial. Telehealth, often in combination with portable specialised devices, facilitates the provision of specialist services in underserved (e.g., rural) communities, enabling specialist consultation without being in the room with the patient [5, 6]. Virtual triaging is assisting in emergency situations; for example, the GoodSAM platform enables emergency medical services to use live streaming to track vital signs including heart rate, respiration rate, and temperature using artificial intelligence [25]. Video triaging has also been successfully used to prevent unnecessary hospital visits for paediatric patients [26, 27].

### 4.1. Limitations

Limitations of this research include the secondary use of clinical data collected primarily for continuity of care and clinical audit. Prioritised ethnicity data is a limitation as, in a country as diverse as New Zealand, service users are likely to identify with more than one ethnic group; this might mean that some ethnic groups are underrepresented, as service users can only identify with one ethnicity. Unfortunately, there are also inaccuracies with Statistics New Zealand and NHI data, especially with Māori ethnicity data [28]. Multiple images can be uploaded by a single service user on one or more occasions meaning this data does not necessarily represent individual service users; however, only 2% of the images were found to be duplicates. It is unknown how many service users were asked to upload an image and did not. This research also does not show whether a service user followed the advice of a Healthline clinician, only the recommended outcome. There is also the issue surrounding anonymous service users and the lack of information surrounding these users, resulting in once again certain ethnic groups being underrepresented. It is, however, a right of Whakarongorau service users to remain anonymous without impacting the outcome of their call. This research does not include individual-level data for service users who did not upload an image to Healthline. A simple linear regression model was used to evaluate the relationship between number of calls and time; therefore, confounding variables were not evaluated.

Future research will investigate whether the introduction of the Māori pathway to Healthline (launched Dec 2022) increased the utilisation of the image upload tool by Māori patients. It will also be necessary to investigate whether Pasifika are choosing not to use the image upload tool or the more likely reason that Pasifika use Healthline less than other New Zealand ethnic groups. In line with previous research findings, Whakarongorau has made a commitment to increasing access to Pasifika. Research surrounding Healthline's new video streaming technology (launched Jan 2023) will be of great interest, especially compared with image technology.

## 5. Conclusion

This study has detailed the monthly trends and demographics of the service users who have uploaded an image as part of the free 24/7 Healthline service. A significant outcome difference is apparent by comparing total Healthline outcomes with image upload outcomes. Service users who did not upload an image had a higher proportion of Emergency Department outcomes than service users who did use the tool. Uptake of the image upload system by clinicians increased with time, where hopefully clinicians saw for themselves the benefit of the service. This image upload tool has shown the potential to decrease stress on Emergency Departments around Aotearoa, New Zealand, through increased lower acuity outcomes. The introduction and utilisation of an image upload system in a telehealth service has the potential to allow for better-informed health decisions by telehealth clinicians at the same free cost to service users.

## Figures and Tables

**Table 1:** Demographics of service users who uploaded an image through Healthline compared to the total Healthline contacts (2021-2022). The acronym “MELAA” stands for “Middle Eastern, Latin American, and African.” See Table T2 in the supplementary material.

Variable	Variable category	Images uploaded	%	Total Healthline contacts	Significance
Gender	Female	22,214	55.5%	61.9%	*p* < 0.0001
	Male	17,615	44.0%	37.2%	*p* < 0.0001
	Other	14	0.0%	0.1%	
	Unknown	—	0.0%	0.8%	*p* < 0.0001

Age	0-12	18,103	45.2%	22.7%	*p* < 0.0001
	13-29	9,529	23.8%	27.5%	*p* < 0.0001
	30-44	6,307	15.7%	21.4%	*p* < 0.0001
	45-59	3,311	8.3%	12.6%	*p* < 0.0001
	60-74	1,760	4.4%	8.7%	*p* < 0.0001
	75+	1,001	2.5%	4.1%	*p* < 0.0001
	Unknown	118	0.3%	3.0%	*p* < 0.0001

Ethnicity	NZ European	23,878	59.6%	57.9%	*p* < 0.0001
	Māori	8,319	20.8%	17.7%	*p* < 0.0001
	Pasifika	2,284	5.7%	5.9%	
	Asian	1,990	5.0%	4.6%	*p* < 0.0001
	MELAA	300	0.7%	0.7%	
	Other	1,957	4.9%	5.4%	*p* < 0.0001
	Unknown	1,317	3.3%	7.9%	*p* < 0.0001

GCH rurality (2022)	Urban 1	10,593	60.9%	61.1%	
	Urban 2	3,107	17.9%	16.4%	*p* < 0.0001
	Rural 1	1,710	9.8%	8.7%	*p* < 0.0001
	Rural 2	642	3.7%	2.8%	*p* < 0.0001
	Rural 3	216	1.2%	0.9%	*p* < 0.0001
	Unknown	1,131	6.5%	10.1%	*p* < 0.0001

## Data Availability

Aggregated data associated with this article is provided in the attached supplementary file.

## Supplementary Materials

Supplementary Materials A supplementary material file is attached. This file includes all of the data used in this research manuscript to create each figure and table (Table T1-T7). This file also includes further results not included in this manuscript (Figure S1 and S2).
