# Supplementary material for: Enhancing Aotearoa, New Zealand's Free Healthline Service through Image Upload Technology
Source: Int J Telemed Appl. 2024 Feb 2;2024:6644580. doi: 10.1155/2024/6644580 (PMC10857879; doi:10.1155/2024/6644580)
Supplement: Supplementary Materials — A supplementary material file is attached. This file includes all of the data used in this research manuscript to create each figure and table (Table T1-T7). This file also includes further results not included in this manuscript (Figure S1 and S2). [file 6644580.f1.zip › Supplementary Material v3.docx]

**Supplementary Material**

**Table T1.** Image Upload Contacts by Month 2021-2022.

| **Month** | **Image Upload Contacts** | **Daily Average** |
| --- | --- | --- |
| Mar-21 | 1,146 | 39.5 |
| Apr-21 | 1,369 | 45.6 |
| May-21 | 1,503 | 48.5 |
| Jun-21 | 1,420 | 47.3 |
| Jul-21 | 1,405 | 45.3 |
| Aug-21 | 1,603 | 51.7 |
| Sep-21 | 1,797 | 59.9 |
| Oct-21 | 1,992 | 64.3 |
| Nov-21 | 1,924 | 64.1 |
| Dec-21 | 2,260 | 72.9 |
| Jan-22 | 2,333 | 75.3 |
| Feb-22 | 1,476 | 52.7 |
| Mar-22 | 1,269 | 40.9 |
| Apr-22 | 1,591 | 53.0 |
| May-22 | 1,665 | 53.7 |
| Jun-22 | 1,719 | 57.3 |
| Jul-22 | 2,039 | 65.8 |
| Aug-22 | 2,294 | 74 |
| Sep-22 | 1,924 | 64.1 |
| Oct-22 | 2,275 | 73.4 |
| Nov-22 | 2,321 | 77.4 |
| Dec-22 | 2,720 | 87.7 |
| **Total** | 40,045 | 59.9 |

**Table T2.** Image Upload Contacts by Ethnic Group (2021-2022) compared to the NZ demographic (2018).^18^ The acronym ‘MELAA’ stands for “Middle Eastern, Latin American and African”.

| **Ethnic Group** | **Image Upload Contacts** | **%** | **NZ demographic (2018)^18^** |
| --- | --- | --- | --- |
| NZ European | 23,878 | 59.6% | 70.2% |
| Māori | 8,319 | 20.8% | 16.5% |
| Pasifika | 2,284 | 5.7% | 8.1% |
| Asian | 1,990 | 5.0% | 15.1% |
| MELAA | 300 | 0.7% | 1.5% |
| Other | 1,957 | 4.9% | 1.2% |
| Unknown | 1318 | 3.3% | - |
| Total | 40,045 | 100% | 100% |

**Table T3.** Image Upload Contacts by Age Group and Year (2021-2022), with proportions.

| **Age Group** | **2021 images uploaded** | **2022 images uploaded** | **Total %** | **Total Healthline Calls (2022)** |
| --- | --- | --- | --- | --- |
| Under 1 | 1,290 | 2,309 | 9.0% | 18,115 (4.8%) |
| 1-2 years | 1,383 | 2,233 | 9.0% | 15,543 (4.1%) |
| 2-5 years | 2,662 | 3,776 | 16.1% | 33,029 (8.7%) |
| 6-12 years | 1,858 | 2,592 | 11.1% | 23,883 (6.3%) |
| 13-19 years | 1,262 | 1,735 | 7.5% | 26,411 (7.0%) |
| 20-24 years | 1,435 | 1,906 | 8.4% | 38,492 (10.1%) |
| 25-29 years | 1,408 | 1,783 | 8.0% | 37,327 (9.8%) |
| 30-34 years | 1,183 | 1,539 | 6.8% | 33,524 (8.8%) |
| 35-39 years | 895 | 1,144 | 5.1% | 24,694 (6.5%) |
| 40-44 years | 578 | 968 | 3.9% | 19,973 (5.3%) |
| 45-49 years | 536 | 706 | 3.1% | 16,742 (4.4%) |
| 50-54 years | 456 | 641 | 2.7% | 16,188 (4.3%) |
| 55-59 years | 398 | 574 | 2.4% | 13,928 (3.7%) |
| 60-64 years | 282 | 462 | 1.9% | 13,058 (3.4%) |
| 65-74 years | 401 | 615 | 2.5% | 20,775 (5.5%) |
| 75-84 years | 231 | 392 | 1.6% | 12,914 (3.4%) |
| 85+ years | 152 | 226 | 0.9% | 4,449 (1.2%) |
| **Total** | 16,410 | 23,601 | 100.0% | 379,571 |

**Table T4.** Image Upload Contacts by New Zealand Urban and Rural Areas in 2022.

| **NZ Area Type** | **Images Uploaded** | **%** | **Total Healthline Calls** | **%** |
| --- | --- | --- | --- | --- |
| Urban-1 | 10,593 | 60.9% | 234,233 | 61.1% |
| Urban-2 | 3,107 | 17.9% | 62,867 | 16.4% |
| Rural-1 | 1,710 | 9.8% | 33,240 | 8.7% |
| Rural-2 | 642 | 3.7% | 10,742 | 2.8% |
| Rural-3 | 216 | 1.2% | 3,515 | 0.9% |
| Unknown | 1,131 | 6.5% | 38,881 | 10.1% |
| **Total** | 17,399 | 100.0% | 383,478 | 100.0% |


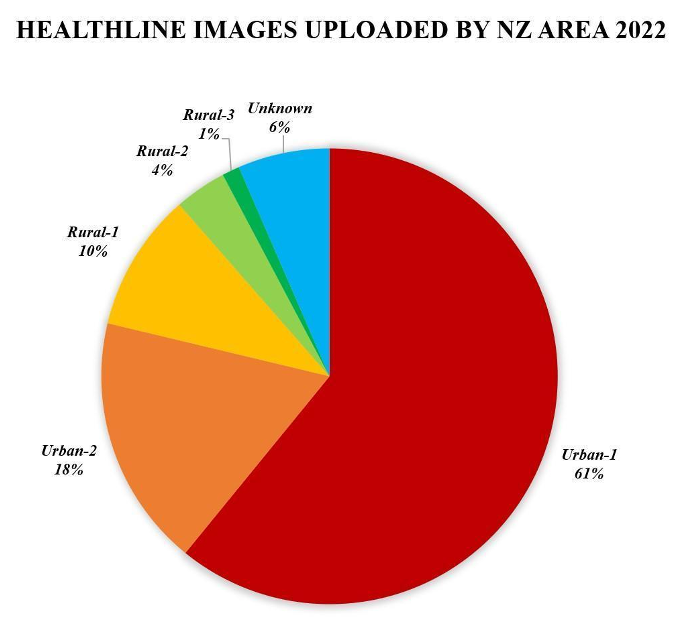


**Figure S1.** Healthline image upload contacts by New Zealand area and whether it is urban or rural (2022). With Urban-1 being the most urban and Rural-3 being the most rural/remote areas.

**Table T5.** New Zealand Urban and Rural Area image upload proportions comparison with the NZ demographic (2018).^18^

| **NZ Area Type** | **Healthline Images Uploaded %** | **NZ Demographic % ^18^** |
| --- | --- | --- |
| Total Urban | 78.7 | 83.7 |
| Total Rural | 14.8 | 16.3 |
| Unknown | 6.5 | - |

**Table T6.** Comparison of Healthline Outcomes for total Healthline calls and the number of image upload contacts (2021-2022).

| **Healthline Outcome** | **Total Healthline Calls** | **%** | **No. of Image Upload Contacts** | **%** |
| --- | --- | --- | --- | --- |
| 111 Emergency | 24,192 | 3.6% | 390 | 1.2% |
| Emergency Department | 66,479 | 9.9% | 2,124 | 6.5% |
| Urgent Care | 113,646 | 16.9% | 7,883 | 24.0% |
| On Call Dr/GP/Other | 14,486 | 2.2% | 1,662 | 5.1% |
| GP | 163,004 | 24.3% | 12,080 | 36.7% |
| Pharmacist | 4,915 | 0.7% | 498 | 1.5% |
| Self Care | 224,212 | 33.4% | 7,550 | 22.9% |
| Other | 60,630 | 9.0% | 713 | 2.2% |
| **Total** | 671,564 | 100.0% | 32,900 | 100.0% |

**Table T7.** The number of clinicians using the image upload service by month with the average number of uploads by each clinician (Mar 2021 - Jun 2023).

| **Month** | **Number of Clinicians using the service** | **Average no. of uploads by each Clinician** |
| --- | --- | --- |
| Mar-21 | 104 | 8.4 |
| Apr-21 | 129 | 8.3 |
| May-21 | 125 | 9.3 |
| Jun-21 | 121 | 9.5 |
| Jul-21 | 121 | 9.2 |
| Aug-21 | 120 | 10.5 |
| Sep-21 | 140 | 9.7 |
| Oct-21 | 148 | 9.7 |
| Nov-21 | 143 | 10.3 |
| Dec-21 | 151 | 11.3 |
| Jan-22 | 145 | 12.0 |
| Feb-22 | 135 | 7.0 |
| Mar-22 | 136 | 5.1 |
| Apr-22 | 142 | 8.3 |
| May-22 | 178 | 7.2 |
| Jun-22 | 287 | 4.6 |
| Jul-22 | 270 | 5.8 |
| Aug-22 | 260 | 6.9 |
| Sep-22 | 269 | 5.5 |
| Oct-22 | 247 | 6.9 |
| Nov-22 | 233 | 7.5 |
| Dec-22 | 239 | 8.2 |
| **Average** | **181.2** | **8.5** |

**
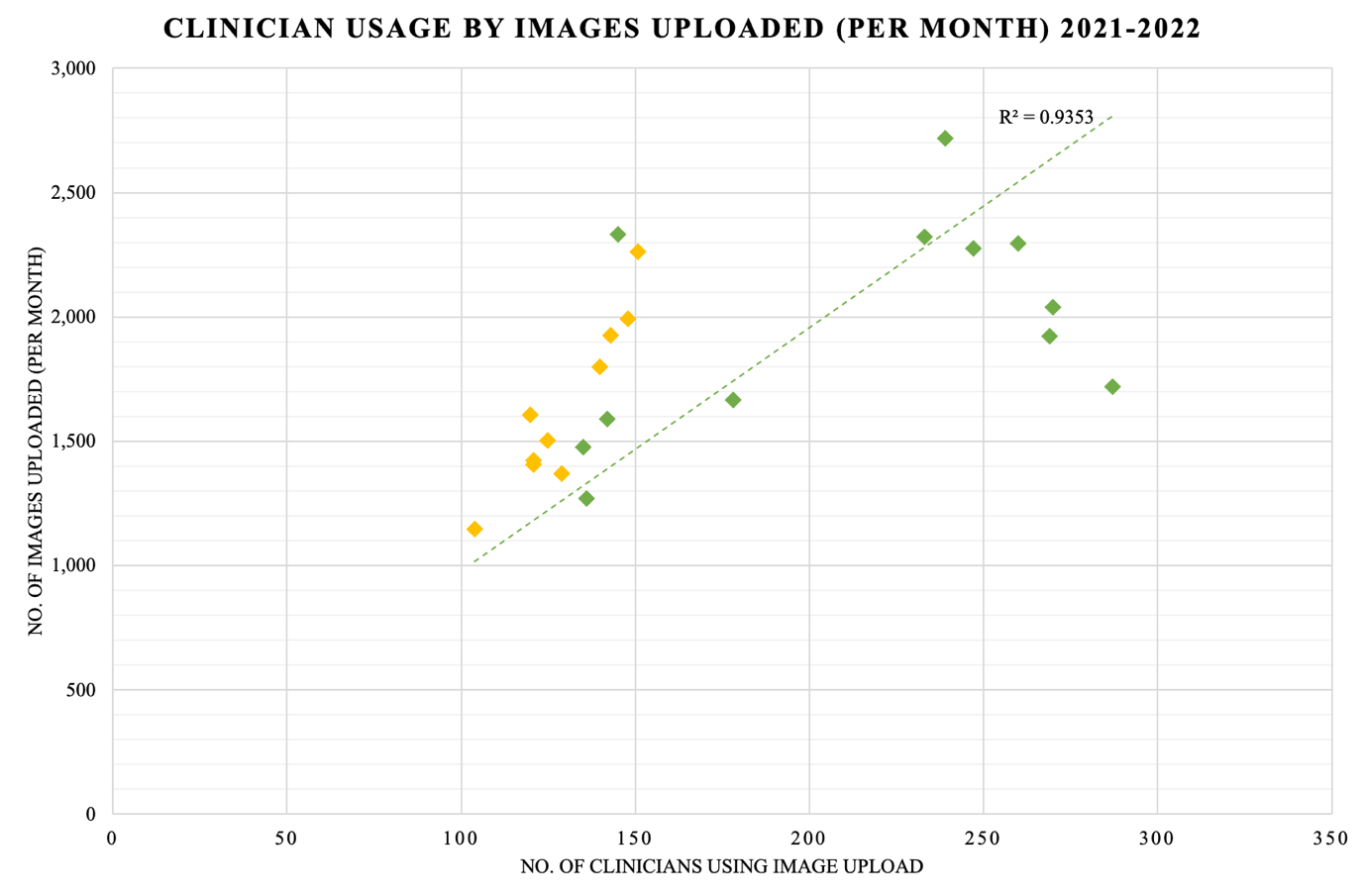
**

**Figure S2.** The number of clinicians using image upload each month is plotted with the number of images uploaded per month (2021-2022). A linear trendline is shown with its respective R^2^ value. Yellow data points represent 2021 data, and green for 2022.
