# Supplementary material for: Enhancing Aotearoa, New Zealand's Free Healthline Service through Image Upload Technology
Source: Int J Telemed Appl. 2024 Feb 2;2024:6644580. doi: 10.1155/2024/6644580 (PMC10857879; doi:10.1155/2024/6644580)
Supplement: Supplementary Materials — A supplementary material file is attached. This file includes all of the data used in this research manuscript to create each figure and table (Table T1-T7). This file also includes further results not included in this manuscript (Figure S1 and S2). [file 6644580.f1.zip › Supplementary Material v3.pdf]

Supplementary Material

Table T1. Image Upload Contacts by Month 2021-2022.

Month	Image Upload Contacts	Daily Average
Mar-21	1,146	39.5
Apr-21	1,369	45.6
May-21	1,503	48.5
Jun-21	1,420	47.3
Jul-21	1,405	45.3
Aug-21	1,603	51.7
Sep-21	1,797	59.9
Oct-21	1,992	64.3
Nov-21	1,924	64.1
Dec-21	2,260	72.9
Jan-22	2,333	75.3
Feb-22	1,476	52.7
Mar-22	1,269	40.9
Apr-22	1,591	53.0
May-22	1,665	53.7
Jun-22	1,719	57.3
Jul-22	2,039	65.8
Aug-22	2,294	74

Sep-22	1,924	64.1
Oct-22	2,275	73.4
Nov-22	2,321	77.4
Dec-22	2,720	87.7
**Total**	40,045	59.9

Table T2. Image Upload Contacts by Ethnic Group (2021-2022) compared to the NZ demographic (2018).[18] The acronym 'MELAA' stands for "Middle Eastern, Latin American and African".

Ethnic Group	Image Upload Contacts	%	NZ demographic (2018)[18]
NZ European	23,878	59.6%	70.2%
Māori	8,319	20.8%	16.5%
Pasifika	2,284	5.7%	8.1%
Asian	1,990	5.0%	15.1%
MELAA	300	0.7%	1.5%
Other	1,957	4.9%	1.2%
Unknown	1318	3.3%	-
Total	40,045	100%	100%

Table T3. Image Upload Contacts by Age Group and Year (2021-2022), with proportions.

Age Group	2021 images uploaded	2022 images uploaded	Total %	Total Healthline Calls (2022)
Under 1	1,290	2,309	9.0%	18,115 (4.8%)
1-2 years	1,383	2,233	9.0%	15,543 (4.1%)
2-5 years	2,662	3,776	16.1%	33,029 (8.7%)
6-12 years	1,858	2,592	11.1%	23,883 (6.3%)
13-19 years	1,262	1,735	7.5%	26,411 (7.0%)
20-24 years	1,435	1,906	8.4%	38,492 (10.1%)
25-29 years	1,408	1,783	8.0%	37,327 (9.8%)
30-34 years	1,183	1,539	6.8%	33,524 (8.8%)
35-39 years	895	1,144	5.1%	24,694 (6.5%)
40-44 years	578	968	3.9%	19,973 (5.3%)
45-49 years	536	706	3.1%	16,742 (4.4%)
50-54 years	456	641	2.7%	16,188 (4.3%)
55-59 years	398	574	2.4%	13,928 (3.7%)
60-64 years	282	462	1.9%	13,058 (3.4%)

65-74 years	401	615	2.5%	20,775 (5.5%)
75-84 years	231	392	1.6%	12,914 (3.4%)
85+ years	152	226	0.9%	4,449 (1.2%)
**Total**	16,410	23,601	100.0%	379,571

Table T4. Image Upload Contacts by New Zealand Urban and Rural Areas in 2022.

NZ Area Type	Images Uploaded	%	Total Healthline Calls	%
Urban-1	10,593	60.9%	234,233	61.1%
Urban-2	3,107	17.9%	62,867	16.4%
Rural-1	1,710	9.8%	33,240	8.7%
Rural-2	642	3.7%	10,742	2.8%
Rural-3	216	1.2%	3,515	0.9%
Unknown	1,131	6.5%	38,881	10.1%
**Total**	17,399	100.0%	383,478	100.0%

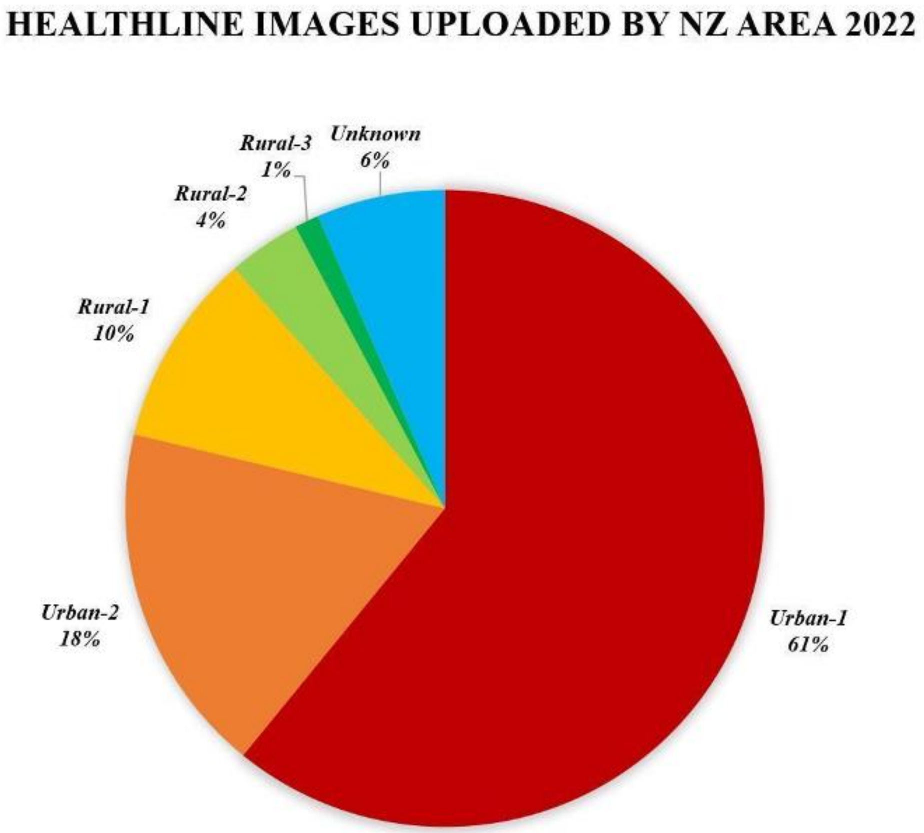

Figure S1. Healthline image upload contacts by New Zealand area and whether it is urban or rural (2022). With Urban-1 being the most urban and Rural-3 being the most rural/remote areas.

Table T5. New Zealand Urban and Rural Area image upload proportions comparison with the NZ demographic (2018).[18]

NZ Area Type	Healthline Images Uploaded %	NZ Demographic % [18]
Total Urban	78.7	83.7
Total Rural	14.8	16.3
Unknown	6.5	-

Table T6. Comparison of Healthline Outcomes for total Healthline calls and the number of image upload contacts (2021-2022).

Healthline Outcome	Total Healthline Calls	%	No. of Image Upload Contacts	%
111 Emergency	24,192	3.6%	390	1.2%
Emergency Department	66,479	9.9%	2,124	6.5%
Urgent Care	113,646	16.9%	7,883	24.0%
On Call Dr/GP/Other	14,486	2.2%	1,662	5.1%
GP	163,004	24.3%	12,080	36.7%
Pharmacist	4,915	0.7%	498	1.5%
Self Care	224,212	33.4%	7,550	22.9%
Other	60,630	9.0%	713	2.2%
**Total**	671,564	100.0%	32,900	100.0%

Table T7. The number of clinicians using the image upload service by month with the average number of uploads by each clinician (Mar 2021 - Jun 2023).

Month	Number of Clinicians using the service	Average no. of uploads by each Clinician
Mar-21	104	8.4
Apr-21	129	8.3
May-21	125	9.3
Jun-21	121	9.5
Jul-21	121	9.2
Aug-21	120	10.5

Sep-21	140	9.7
Oct-21	148	9.7
Nov-21	143	10.3
Dec-21	151	11.3
Jan-22	145	12.0
Feb-22	135	7.0
Mar-22	136	5.1
Apr-22	142	8.3
May-22	178	7.2
Jun-22	287	4.6
Jul-22	270	5.8
Aug-22	260	6.9
Sep-22	269	5.5
Oct-22	247	6.9
Nov-22	233	7.5
Dec-22	239	8.2
**Average**	**181.2**	**8.5**

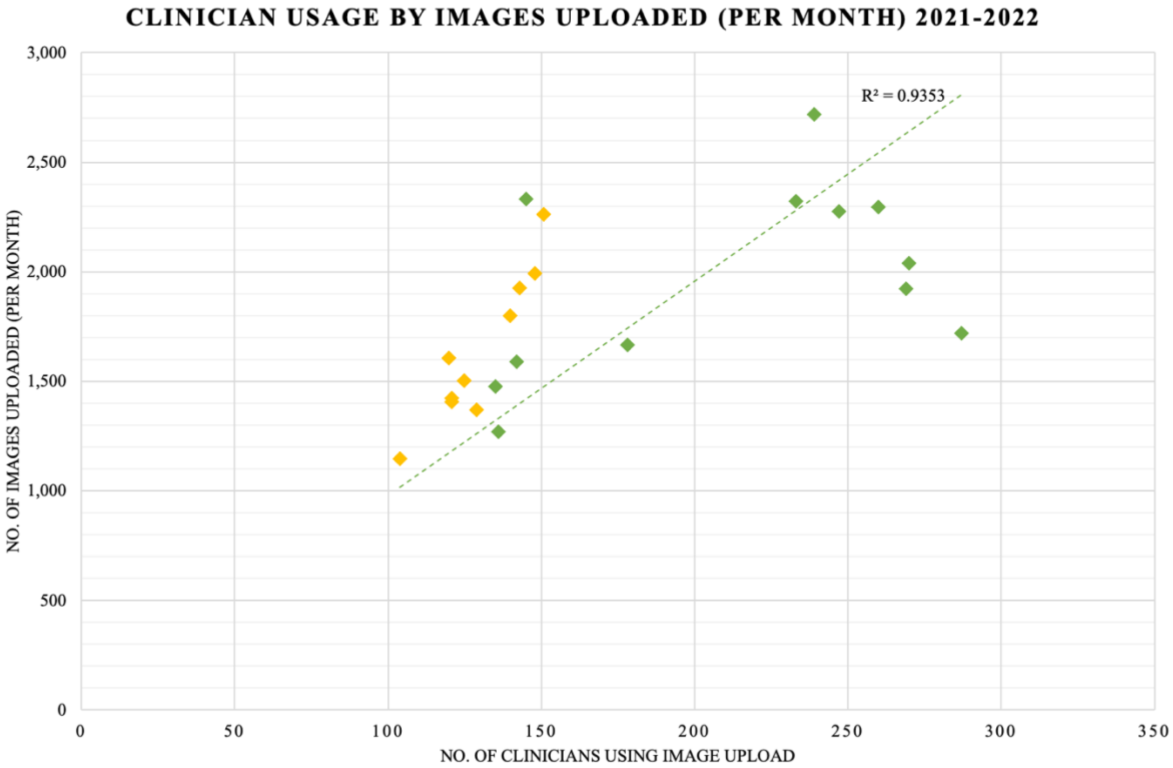

Figure S2. The number of clinicians using image upload each month is plotted with the number of images uploaded per month (2021-2022). A linear trendline is shown with its respective R^2 value. Yellow data points represent 2021 data, and green for 2022.
